# Beneficial Insects Deliver Plant Growth-Promoting Bacterial Endophytes between Tomato Plants

**DOI:** 10.3390/microorganisms9061294

**Published:** 2021-06-14

**Authors:** Nikoletta Galambos, Stéphane Compant, Felix Wäckers, Angela Sessitsch, Gianfranco Anfora, Valerio Mazzoni, Ilaria Pertot, Michele Perazzolli

**Affiliations:** 1 Research and Innovation Centre, Department of Sustainable Ecosystems and Bioresources, Fondazione Edmund Mach, Via E. Mach 1, 38098 San Michele all’Adige, Italy; nikoletta.galambos@fmach.it (N.G.); gianfranco.anfora@unitn.it (G.A.); valerio.mazzoni@fmach.it (V.M.); ilaria.pertot@unitn.it (I.P.); 2 Department of Civil, Environmental and Mechanical Engineering, University of Trento, via Mesiano 77, 38123 Trento, Italy; 3 Biobest NV, Isle Velden 18, 2260 Westerlo, Belgium; Felix.Wackers@biobestgroup.com; 4 Center for Health and Bioresources, AIT Austrian Institute of Technology, Konrad Lorenz Straβe 24, 3430 Tulln, Austria; Stephane.Compant@ait.ac.at (S.C.); Angela.Sessitsch@ait.ac.at (A.S.); 5 Center Agriculture Food Environment (C3A), University of Trento, via E. Mach 1, 38098 San Michele all’Adige, Italy

**Keywords:** plant growth-promoting bacterial endophytes, beneficial mirids, *Macrolophus pygmaeus*, *Nesidiocoris tenuis*

## Abstract

Beneficial insects and mites, including generalist predators of the family Miridae, are widely used in biocontrol programs against many crop pests, such as whiteflies, aphids, lepidopterans and mites. Mirid predators frequently complement their carnivore diet by feeding plant sap with their piercing–sucking mouthparts. This implies that mirids may act as vectors of phytopathogenic and beneficial microorganisms, such as plant growth-promoting bacterial endophytes. This work aimed at understanding the role of two beneficial mirids (*Macrolophus pygmaeus* and *Nesidiocoris tenuis*) in the acquisition and transmission of two plant growth-promoting bacteria, *Paraburkholderia phytofirmans* strain PsJN (PsJN) and *Enterobacter* sp. strain 32A (32A). Both bacterial strains were detected on the epicuticle and internal body of both mirids at the end of the mirid-mediated transmission. Moreover, both mirids were able to transmit PsJN and 32A between tomato plants and these bacterial strains could be re-isolated from tomato shoots after mirid-mediated transmission. In particular, PsJN and 32A endophytically colonised tomato plants and moved from the shoots to roots after mirid-mediated transmission. In conclusion, this study provided novel evidence for the acquisition and transmission of plant growth-promoting bacterial endophytes by beneficial mirids.

## 1. Introduction

Beneficial insects (e.g., hymenopteran parasitoids, bumblebees, hoverflies, lacewings, ladybugs and mirids) provide vital services in agricultural ecosystems, such as pollination and natural pest control [1,2]. Among insect predators, the mirids *Macrolophus pygmaeus* (Rambur) and *Nesidiocoris tenuis* (Reuter) (Hemiptera: Miridae) are widely used for the biocontrol of whiteflies, aphids and moths, among others [3,4,5,6]. Tomato can be damaged by phytophagous arthropods worldwide [7], both under field and greenhouse conditions [8]. For example, leafminers (e.g., *Tuta absoluta* and the dipteran *Liriomyza* spp.) and phytophagous mites (e.g., *Tetranychus* sp. and *Aculops lycopersici*) reduce the photosynthetic area and the latter also increase plant transpiration and alter the water balance [9]. Aphids (e.g., *Myzus persicae, Aphis* sp.), thrips (e.g., *Frankliniella occidentalis* and *Thrips tabaci*) and whitefly species (e.g., *Bemisia tabaci* and *Trialeurodes vaporariorum*) cause direct and indirect damage due to virus transmission [9]. To counteract these pests, the generalist mirids (e.g., *M. pygmaeus* and *N. tenuis*) are increasingly used in many European and adjacent countries [7], thanks to their ability to rapidly colonise tomato plants and to establish stable colonies early in the growing season [7,10,11,12].

*Macrolophus pygmaeus* and *N. tenuis* have piercing and sucking mouthparts that contain two channels: one to pump salivary fluid into plant tissues and the other to suck sap fluids from the host [13]. These two species frequently complement their carnivore diet with plant sap feeding [14,15], which could also lead to slight yield losses [16,17]. For example, *M. pygmaeus* feeds mainly on the mesophyll of leaves, stems and fruits, and high population densities caused fruit damage when plants are infected with pepino mosaic virus (PepMV) [18]. On the other hand, *N. tenuis* feeds mainly within the vascular semi-ring of tomato plants [19,20], and high population densities cause necrotic rings in both the leaves and flower petioles, and whitish halos on the fruits, independently of the presence of PepMV [21,22].

Mouthpart morphology and trophic behaviour suggest possible exchange of microorganisms between the insects and host plants, as has been demonstrated in many hemipteran species [23]. Mirids are generally considered negligible vectors of plant pathogens [24], but *Erwinia amylovora* [25] and *Lonsdalea quercina* pv. *lupinicola* [26] can be transmitted by herbivorous *Lygus* spp. Likewise, *Pantoea* spp. and *Serratia marcescens* can be transmitted by *Lygus hesperus* [27] and velvet tobacco mottle virus can be transmitted by *Cyrtopeltis nicotianae* [24,28]. In the case of *M. pygmaeus* and *N. tenuis*, they can both transmit parietaria mottle virus from *Parietaria officinalis* to tomato plants [29]. Insect-mediated disease transmission can occur by direct feeding on infected plants [28], through excretion products [30] or by physical contact [25], suggesting multiple mechanisms of possible transmission of plant-associated microorganisms by *M. pygmaeus* and *N. tenuis*. In addition to vectoring pathogens, insects can also transmit beneficial microbes as proved in the case of the leafhopper *Scaphoideus titanus* (Hemiptera: Cicadellidae), which was shown to transfer non-phytopathogenic bacterial communities between grapevine plants [31]. In this regard, no information is available for other insect/plant systems and in particular on the potential role of beneficial mirids in transmitting plant-associated beneficial microorganisms.

A multitude of bacteria, including those with beneficial effects for the plant host, live in the plant rhizosphere thanks to the presence of root exudates [32]. Among them, the so called plant growth-promoting bacteria (PGPB) can improve plant growth and increase nutrient supply, including nitrogen, phosphorus and iron [33]. Some PGPB can also colonise the internal tissues of numerous plant species. These beneficial endophytes can positively influence plant growth through various mechanisms, such as the production of hormones, improvement of nutrient uptake and protection against biotic or abiotic stresses [34]. For example, the beneficial endophyte *Paraburkholderia phytofirmans* PsJN (PsJN), previously isolated from surface-sterilised onion roots [35] and classified as *Pseudomonas* and then *Burkholderia* [35,36], was able to promote plant growth [37,38,39] and upregulate the genes related to protein metabolism, transcription, transport, defence pathways, signal transduction and hormone metabolism in tomato [39]. Similarly, *Enterobacter* sp. 32A (32A) was isolated from the grapevine endosphere [40] and promoted plant growth [39], activating a complex transcriptional reprogramming in tomato [39] and defence pathways in grapevine [41] plants. Moreover, 32A inhibited the growth of plant pathogens (e.g., *Botrytis cinerea, Botryosphaeria dothidea* and *B. obtusa*) [40] suggesting the use of PGPB to improve plant growth and health [39].

In order to understand if beneficial mirids are able to acquire and transmit beneficial endophytes between tomato plants, we designed experiments to prove if PGPBs can be acquired from tomato plants previously inoculated and transmitted to non-inoculated plants by beneficial mirids. Moreover, the ability of PGPB to reduce feeding damage of mirids was addressed, when tomato plants were offered as the sole food source. This knowledge could greatly contribute to the development of combined approaches in biocontrol and biofertilisation.

## 2. Material and Methods

### 2.1. Bacterial Strains and Inoculum Preparation

Two bacterial strains were used, namely, *Paraburkholderia phytofirmans* PsJN (PsJN), isolated from surface-sterilised onion roots [35], and *Enterobacter* sp. 32A (32A), isolated from a grapevine endosphere [40], which were able to promote tomato growth [39]. Bacterial strains were long-term stored in 80% glycerol at –80 °C. To obtain the inoculum, they were grown in 5 mL nutrient broth (NB) in sterile 15 mL tubes at 25 °C for 24 h under orbital shaking at 220 rpm. Bacterial cells were collected by centrifugation at 3500× *g* for 10 min and washed twice with sterile 10 mM MgSO_4_. Bacterial cells were then suspended in sterile 10 mM MgSO_4_ and the bacterial suspension was adjusted to 1.0 × 10^7^ colony forming units (CFU) per unit of volume (CFU mL^−1^) based on an optical density conversion at 600 nm (OD_600_) optimised for each strain (OD_600_ 0.1 corresponded to 2.34 × 10^8^ CFU mL^−1^ for PsJN and to 6.90 × 10^7^ CFU mL^−1^ for 32A) [39].

### 2.2. Tomato Seed Inoculation and Growth Conditions

**Figure 1:**
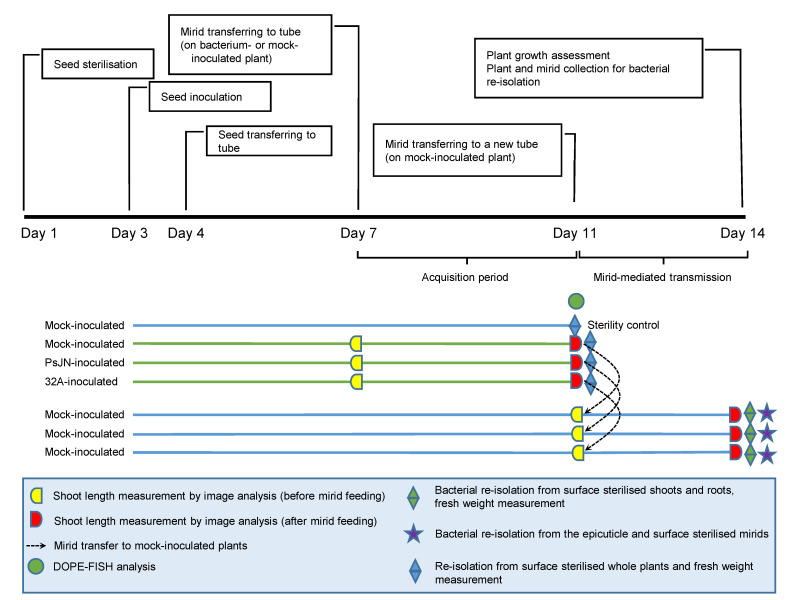
Description of the experiment. Day 1, surface-disinfected seeds were transferred to Petri dishes containing 1% water agar and were incubated in a growth chamber to allow seed germination. Day 3, seeds were treated with sterile MgSO_4_ (mock-inoculated) or inoculated with a bacterial suspension of *Paraburkholderia phytofirmans* PsJN (PsJN) or *Enterobacter* sp. 32A (32A) by overnight incubation in the growth chamber. Day 4, germinated seeds with the same root length were selected and each seed was transferred to a sterile glass tube containing half-strength Hoagland. Day 7, a freshly emerged mirid adult (*Macrolophus pygmaeus* or *Nesidiocoris tenuis*) was placed in each glass tube containing a tomato plant that was either mock-inoculated or inoculated with PsJN or 32A. Before transferring the mirid in the glass tube, shoot length was measured by image analysis. Tubes were incubated in the growth chamber in order to allow mirids to feed on tomato plants (acquisition period). Day 11, for double labelling of the oligonucleotide probes for fluorescence in situ hybridisation (DOPE-FISH) analysis, mirids were collected at the end of the acquisition period on mock-inoculated plants or plants inoculated with PsJN or 32A, shoot length was measured by image analysis, and whole plants were collected for bacterial re-isolation and fresh weight assessment. Each mirid was transferred to a new glass tube containing a mock-inoculated tomato plant and incubated to allow the mirids to feed on the tomato plants (mirid-mediated transmission), and then shoot length was measured. Day 14, mirids and plants after mirid-mediated transmission were collected for bacterial re-isolation, and the tomato shoot length and fresh weight were assessed.

Seeds of *S. lycopersicum* L. cv. Moneymaker (Justseed, Wrexham, UK) were disinfected with 70% ethanol for 1 min and 2% sodium hypochlorite plus 0.02% Tween 20 for 5 min in a 50 mL tube with vigorous shaking and finally washed three times (3 min each) with sterile distilled water (Day 1; Figure 1) [39]. Surface-disinfected seeds (120 seeds) were transferred to Petri dishes (100 mm diameter, 20 seeds for each dish) containing 1% water agar (Thermo Fisher Scientific, Waltham, MA, USA) and then incubated at 21 ± 1 °C in a growth chamber (Bertagnin, Bologna, Italy) to allow seed germination. Seeds were treated with 1 mL of sterile 10 mM MgSO_4_ (mock-inoculated) or inoculated with 1 mL of the bacterial suspension (bacterium-inoculated) using the respective bacterial strain (1 × 10^7^ CFU mL^−1^ PsJN or 32A) by overnight incubation in the growth chamber (seed-inoculated plants) (Day 3) [39]. Germinated seeds with the same root length (5 mm) were selected and each seed was transferred to a sterile 95 mL glass tube (Artiglass, Padova, Italy) containing 15 mL solid (7 g L^−1^ agar) half-strength Hoagland (Day 4) [39]. Plants were incubated in a growth chamber at 21 ± 1 °C with a 16/8 light/dark photoperiod.

### 2.3. Mirid-Mediated Bacterial Transmission Assays

Beneficial mirids (*M. pygmaeus* and *N. tenuis*) were provided by Biobest NV (Westerlo, Belgium). Mirids had been mass-reared for several generations on tomato (*S. lycopersicum* L. cv. Moneymaker) under greenhouse conditions at 28 ± 5 °C with a 16/8 light/dark photoperiod and were supplied with eggs of *Ephestia kuehniella* (Biobest Group NV, Westerlo, Belgium) as food source. To obtain freshly emerged adults, fifth instar nymphs were individually transferred to 100 mL plastic cups, covered with mesh, containing bean pods and *E. kuehniella* eggs [42]. A freshly emerged mirid adult was placed in each glass tube containing a tomato plant that was either mock-inoculated or inoculated with PsJN or 32A (Day 7; Figure 1). Before transferring the mirid into the glass tube, plant shoot length was measured by image analysis using ImageJ version 1.50e [43]. Plants were incubated in the growth chamber at 21 ± 1 °C with a 16/8 light/dark photoperiod, in order to allow mirids to feed on tomato plants (acquisition period). At the end of the acquisition period (Day 11), the shoot length was measured by image analysis, and five mirids for each treatment were sampled for double labelling of oligonucleotide probes for fluorescence in situ hybridisation (DOPE-FISH) analysis and whole plants were collected for bacterial re-isolation (Day 11). Each remaining mirid was transferred to a new glass tube containing a mock-inoculated tomato plant and incubated in the growth chamber to allow mirids to feed on the tomato plants (mirid-mediated transmission). Shoot length was measured by image analysis at the beginning (Day 11) and at the end (Day 14) of the mirid-mediated transmission. After mirid-mediated transmission (Day 14), mirids and plants were collected for bacterial re-isolation, the shoot length and the fresh weight were assessed. In addition, three glass tubes containing mock-inoculated plants were kept mirid-free for sterility control. Before transferring mirids into glass tubes, the surface of the half-strength Hoagland was overlaid with 1 mL of sterile melted paraffin (Sigma-Aldrich, Merck, Darmstadt, Germany), in order to avoid contact of the mirids with either the tomato roots or the growth medium and to prevent contaminations [31].

### 2.4. Bacterial Re-Isolation from Tomato Plants and Mirids

Seed-inoculated plants and plants after mirid-mediated transmission were collected at Day 11 and Day 14, respectively (Figure 1). Mirids were collected at Day 14 for bacterial re-isolation and each mirid was washed with 200 µL distilled water by vigorous vortexing in a 2 mL tube to collect the majority of bacteria adhering to the mirid epicuticle [31]. Each plant was surface-disinfected in a 50 mL tube and each mirid was surface-disinfected in a 2 mL tube with 70% ethanol for 1 min, 2% sodium hypochlorite for 1.5 min, followed by 70% ethanol for 1 min [39]. Surface-disinfected plants, or mirids, were washed three times with distilled water (3 min each). After surface disinfection, each seed-inoculated plant was placed in a 2 mL tube, while the shoots and roots of plants after mirid-mediated transmission were cut and separately placed in 2 mL-tubes. Each plant or shoot was ground in 500 µL potassium phosphate buffer (1 mM, pH 7) in a mixer-mill disruptor (MM 400, Retsch, Haan, Germany) at 25 Hz for 2 min [39]. Conversely, each root or mirid was ground in 200 µL potassium phosphate buffer (1 mM, pH 7) in the mixer-mill disruptor (MM 400, Retsch). Each suspension was serially diluted and 10 µL aliquots were plated in triplicates on nutrient agar (NA). Aliquots of the last washing solution were plated as the control of surface disinfection. After incubation at 25 °C for 72 h, colonisation intensity was calculated as CFU values of bacteria per unit of plant fresh weight (CFU g^−1^), per unit of mirid (CFU mirid^−1^) or per unit of root (CFU root^−1^). Successful bacterial colonisation was calculated as the percentage of positive samples (%), such as seed-inoculated plants, shoots or roots after mirid-mediated transmission, mirid epicuticle or internal body with any amount of the respective bacteria present over the total number of samples, in order to calculate the colonisation intensity after successful mirid-mediated transmission (CFU g^−1^, CFU mirid^−1^ or CFU root^−1^) on positive samples [44]. Nine replicates (seed-inoculated whole plants, shoots and roots after mirid-mediated transmission and mirids) were analysed for each treatment and the experiment was carried out twice.

PsJN and 32A colonies were recognised according to colony morphology and 16S rRNA gene sequencing. In particular, colony morphology (colour, elevation, form and margin) of each bacterial isolate was compared with that of PsJN (white colony with raised elevation, circular and entire margin) and 32A (yellowish colony with raised elevation, circular and entire margin) grown on NA medium. Only a very few morphologies of bacterial colonies were found and no PsJN- or 32A-like colonies were re-isolated from the mock-inoculated plants and from mirids that feed on mock-inoculated plants, indicating the very rare presence of indigenous culturable bacteria. Moreover, no colonies were detected in surface-disinfected mock-inoculated plants that were kept mirid-free for sterility control. For bacterial identification by sequencing, three representative isolates were selected for each experiment and sample type (seed-inoculated whole plants, shoots and roots after mirid-mediated transmission, bacteria adhering to the mirid epicuticle, bacteria in the mirid internal body). Briefly, a loopful of pure colonies were suspended into 1.5 mL tubes containing sterile isotonic solution (0.85% NaCl in distilled water) and mixed with a vortex. Subsequently, the tubes were centrifuged for 2 min at 10,000× *g*. Then, the pellet was suspended in 100 µL 0.05 M NaOH and incubated at 95 °C for 15 min. After a centrifugation of 2 min at 10,000× *g*, the supernatant was used as DNA template [45]. Amplicons of the 16S rRNA gene were obtained with PCR using the 27-forward (5′-AGAGTTTGATCCTGGCTCAG-3′) and 1492-reverse (5′GGTTACCTTGTTACGACTT-3′) primer. PCR products were generated by amplifying 5 µL DNA with 0.1 µM of each primer, 12.5 µL Go Taq Green Master Mix (Promega GmbH, Mannheim, Germany) and 12 µL sterile deionized water. The amplification protocol consisted of denaturation at 94 °C for 5 min, followed by 35 cycles at 94 °C for 1 min, 55 °C for 1 min and 72 °C for 1 min, followed by a final extension at 72 °C for 10 min in a thermal cycler (Biometra GmbH, Göttingen, Germany). Amplicons were purified by ExoProstar Kit (Illustra, Merck) and sequenced at the Sequencing and Genotyping Platform at Fondazione Edmund Mach. The resulting nucleotide sequences were compared to known sequences deposited in the National Center for Biotechnology Information (NCBI) database (https://www.ncbi.nlm.nih.gov/nucleotide/, 1 January 2021) using BLASTN (Basic Local Alignment Tool) and aligned with the original sequences using the programme Mega X version 10.1.1 [46], in order to confirm the PsJN and 32A identity.

### 2.5. Fluorescence In Situ Hybridisation Using Double Labelling of Oligonucleotide Probes

DOPE-FISH analysis was performed on mirids that fed on mock-inoculated plants and plants inoculated with PsJN or 32A (Day 11, Figure 1). Samples were fixed in a 4% paraformaldehyde in phosphate-buffered saline (PBS) solution at 4 °C for five hours and were rinsed three times with 1 × PBS, as previously reported [47]. Mirids were dehydrated in increasing concentrations of ethanol solution (25, 50, 75 and 99%; 20 min each step) and stored at 4 °C. DOPE-FISH was carried out using probes from Eurofins (Germany) labelled at both the 5′ and 3′ positions. The probe mixture targeting eubacteria was composed of EUB338, EUB338II and EUB338III (EUBmix), coupled with a Cy3 fluorochrome and Bphyt probe targeting the 23S rRNA gene of PsJN coupled with Cy5 [48,49,50]. For 32A, the EUBmix and Gam42a probe targeting the 23S rRNA gene of 32A coupled with Cy5 was used [51]. The NONEUB probe coupled with Cy3 or Cy5 was used independently as the negative control [52]. Fluorescent in situ hybridisation was carried out in sterile 1.5 mL tubes at 46 °C for 2 h in the dark with 60 µL hybridisation buffer for PsJN (containing 0.9 M NaCl; 0.02 M Tris HCl, 0.01% SDS, 10% formamide and 5 ng µL^−1^ of each probe) and with 60 µL hybridisation buffer for 32A (containing 0.9 M NaCl; 0.02 M Tris HCl, 0.01% SDS, 35% formamide and 5 ng µL^−1^ of each probe). Washing was conducted at 48 °C for 30 min with a pre-warmed post-FISH solution containing 0.02 M Tris HCl, 0.01% SDS, NaCl and EDTA at a concentration corresponding to the formamide concentration. Samples were then rinsed with distilled water before overnight air-drying in the dark. Samples were observed under a confocal microscope (Olympus Fluoview FV1000 with multiline laser FV5-LAMAR-2 and HeNe (G) laser FV10-LAHEG230-2). Pictures were taken at 405, 488 and 633 nm wavelengths with the Cy3 signal assigned as green and Cy5 as red. Pictures were analysed using Imaris 8 software (BITPLANE, Belfast, United Kingdom). Z-stacks were used to generate whole-stack pictures. Five replicates (mirids) were analysed for each treatment and representative pictures were selected. Pictures were cropped and the light/contrast balance improved in the post process.

### 2.6. Statistical Analysis

All experiments were carried out twice and data were analysed with Past 3.26 software [53]. After validating the data for normal distribution (Shapiro-Wilk test, *p* > 0.05) and variance homogeneity of the data (Levene’s tests, *p* > 0.05), each experiment was analysed singularly and a two-way analysis of variance (ANOVA) was performed to assess the null hypothesis (i.e., non-significant differences between the two experiments, *p* > 0.05). Data from the two repeated experiments were pooled and significant differences among treatments were assessed with the Student’s *t*-test (*p* ≤ 0.05) and the Tukey’ test (*p* ≤ 0.05) in case of pairwise and multiple comparisons, respectively. The CFU values of the bacterial re-isolation were Log_10_-transformed. If normal distribution (Shapiro-Wilk test, *p* ≤ 0.05) or variance homogeneity (Levene’s tests, *p* ≤ 0.05) conditions were not satisfied, the Mann–Whitney test (*p* ≤ 0.05) was used to assess for significant differences in case of pairwise comparisons.

## 3. Results

### 3.1. Beneficial Mirids Acquire Endophytic Bacteria by Feeding on Tomato Plants

**Figure 2:**
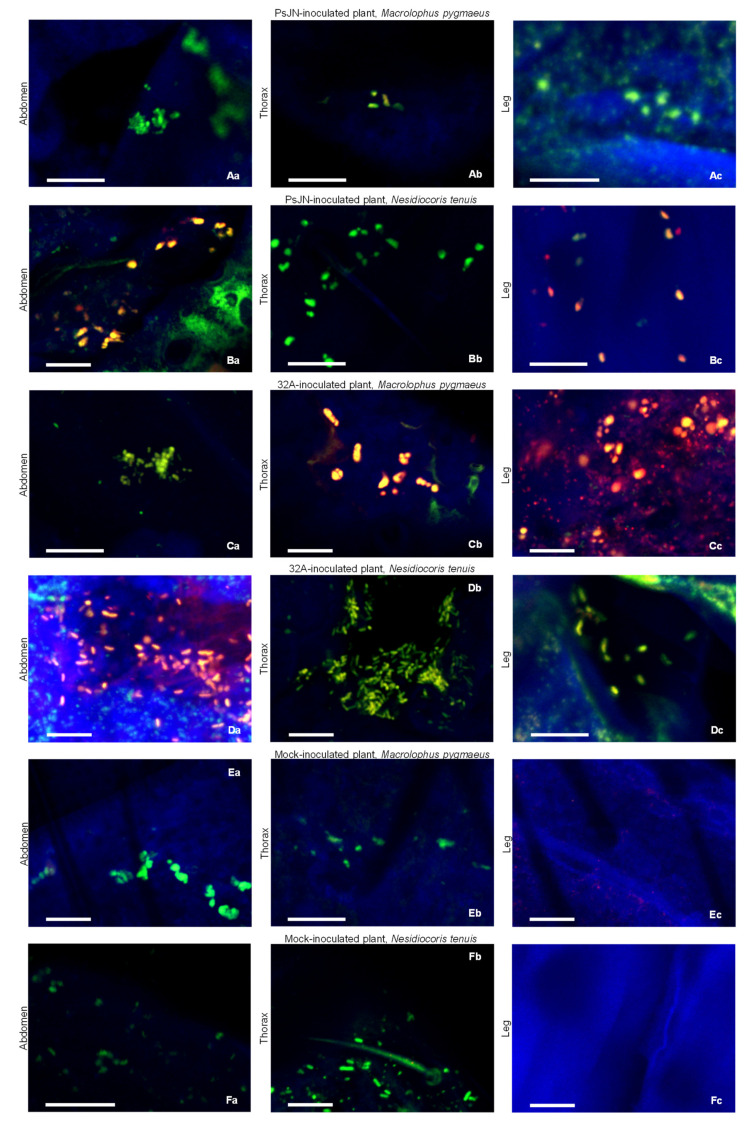
Location of endophytic bacterial strains on mirids. *Macrolophus pygmaeus* (**A**,**C**,**E**) and *Nesidiocoris tenuis* (**B**,**D**,**F**) abdomen (**a**), thorax (**b**) and leg (**c**) samples were collected at the end of the acquisition period (Day 11) on plants inoculated with *Paraburkholderia phytofirmans* PsJN (PsJN) (**A**,**B**) or *Enterobacter* sp. 32A (32A) (**C**,**D**) or mock-inoculated plants (**E**,**F**). PsJN cells were hybridised with the EUBmix and Bphyt probes (**A**,**B**) and 32A cells were hybridised with the EUBmix and Gam42a probes (**C**,**D**). Mirids fed on mock-inoculated plants (**E**,**F**) were hybridised with the EUBmix and Gam42a probes. Five replicates (mirids) were analysed for each treatment and representative pictures were selected. Bars correspond to 10 µm.

To characterise the acquisition of endophytic bacterial strains by beneficial mirids, the DOPE-FISH analysis was carried out on mirids at the end of the acquisition period on bacterium- and mock-inoculated plants (Day 11). Yellow fluorescent PsJN (Figure 2A,B) and 32A (Figure 2C,D) single cells, aggregates and micro-colonies were found on the abdomen, thorax and legs of *M. pygmaeus* and *N. tenuis* that fed on bacterium-inoculated plants. Conversely, only some native bacteria were present on mirids fed on mock-inoculated plants (Figure 2E,F). The NONEUB probe was used as a negative probe not targeting bacterial sequences and only a few green/blue-cyan fluorescent microbes could be detected on mirids fed on mock-, PsJN- and 32A-inoculated plants, as an indicator of the rare presence of indigenous microorganisms (Figure S1).

**Figure 3:**
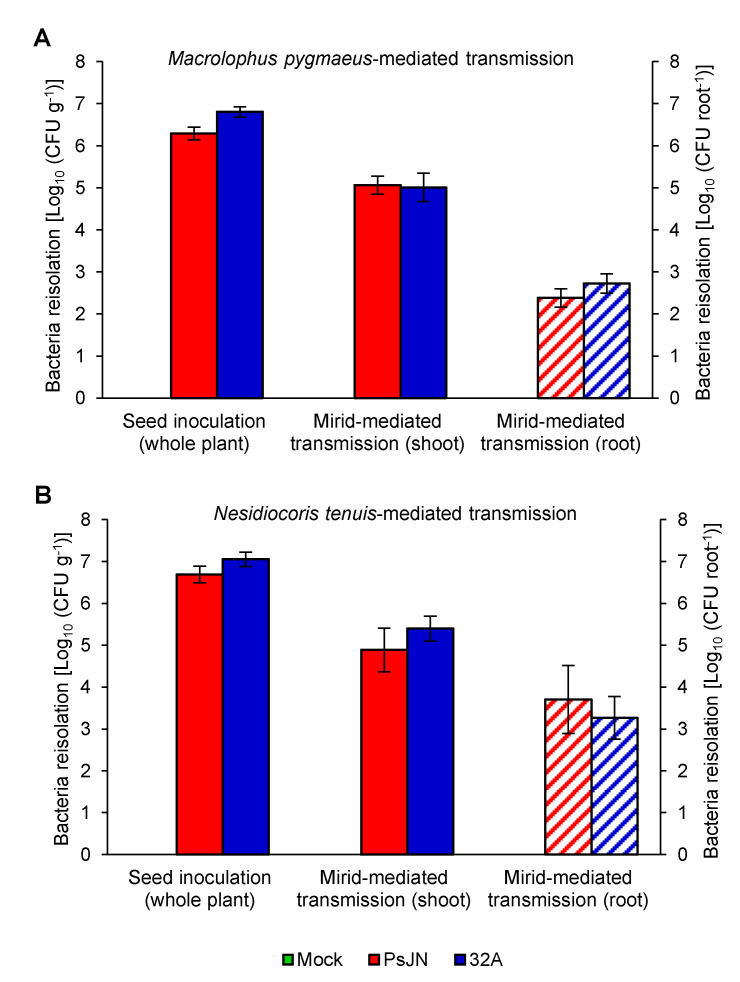
Quantification of endophytic bacterial strains in tomato plants. Bacterial re-isolation was carried out from seed-inoculated whole plants at the end of the acquisition period (Day 11) and from shoots or roots of plants at the end of the mirid-mediated transmission (Day 14) with *Macrolophus pygmaeus* (**A**) or *Nesidiocoris tenuis* (**B**). The quantity of re-isolated bacteria are expressed as colony forming units (CFU) per gram of fresh weight of the whole plant (CFU g^−1^) and plant shoot (CFU g^−1^), or as CFU for each plant root (CFU root^−1^) of mock-inoculated plants (Mock, green) and plants inoculated with *Paraburkholderia phytofirmans* PsJN (PsJN, red) or *Enterobacter* sp. 32A (32A, blue). The two-way analysis of variance showed no significant differences between the two experimental repetitions (*p* > 0.05) and data from the two experiments were pooled. Mean and standard error values for positive samples and at least nine replicates (plants) are presented for each treatment. For each treatment, no significant differences were found in the pairwise comparisons between PsJN- and 32A-inoculated samples, according to the Mann–Whitney test (*p* ≤ 0.05). Neither PsJN nor 32A bacterial colonies were isolated from the mock-inoculated samples.

**Figure 4:**
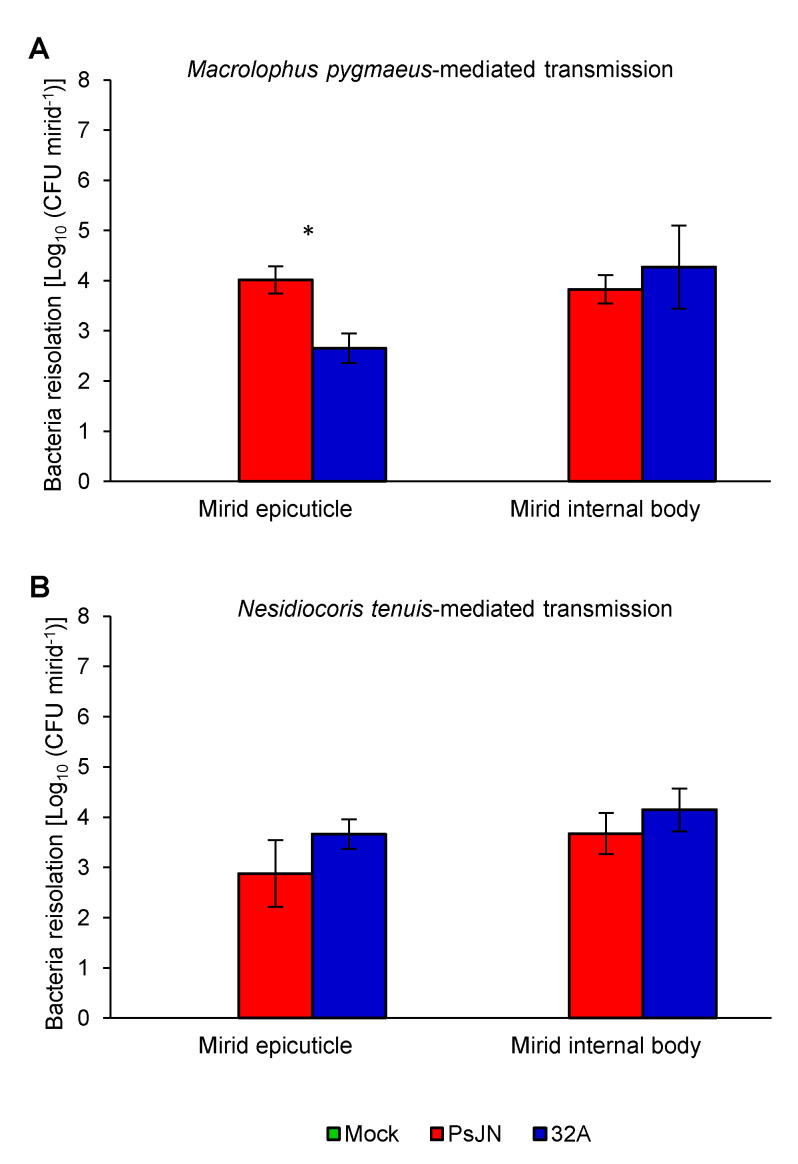
Quantification of endophytic bacterial strains on the mirid epicuticle and internal body. Bacterial re-isolation was carried out from a mirid washing suspension to collect bacteria adhering to the mirid epicuticle or from tissue grinding of surface-disinfected mirids to collect bacteria in the mirid internal body at the end of the mirid-mediated transmission (Day 14) with *Macrolophus pygmaeus* (**A**) or *Nesidiocoris tenuis* (**B**). The quantity of re-isolated bacteria is expressed as the colony forming units per mirid (CFU mirid^−1^) fed on mock-inoculated plants (Mock, green) and plants inoculated with *Paraburkholderia phytofirmans* PsJN (PsJN, red) or *Enterobacter* sp. 32A (32A, blue). The two-way analysis of variance showed no significant differences between the two experimental repetitions (*p* > 0.05) and data from the two experiments were pooled. Mean and standard error values for positive samples and at least nine replicates (mirids) are presented for each treatment. Asterisks indicate significant differences in the pairwise comparisons between the PsJN- and 32A-inoculated samples, according to the Mann–Whitney test (*p* ≤ 0.05). Neither PsJN nor 32A bacterial colonies were isolated from mirids fed on the mock-inoculated plants.

Tomato plants were colonised by endophytic bacterial strains, and PsJN and 32A were re-isolated at the end of the acquisition period (Day 11) from 100% of the seed-inoculated plants, with a colonisation intensity of 3.44 × 10^6^ and 8.81 × 10^6^ CFU g^−1^, respectively (Figure 3A,B). At the end of the mirid-mediated transmission (Day 14), 95.7% and 52.4% of the internal body of *M. pygmaeus* showed a PsJN and 32A colonisation intensity of 6.73 × 10^3^ and 1.86 × 10^4^ CFU mirid^−1^, respectively (Figure 4A). Meanwhile, 69.6% and 81.8% of the *M. pygmaeus* epicuticle showed a PsJN and 32A colonisation intensity of 1.03 × 10^4^ and 4.50 × 10^2^ CFU mirid^−1^, respectively (Figure 4A). Moreover, PsJN and 32A colonisation intensity was 4.71 × 10^3^ and 1.40 × 10^4^ CFU mirid^−1^ in 47.1% and 89.5% of the *N. tenuis* internal body or 7.56 × 10^2^ and 4.58 × 10^3^ CFU mirid^−1^ in 23.5% and 75.0% of the *N. tenuis* epicuticle, respectively (Figure 4B). However, PsJN and 32A were not detected in surface-disinfected mock-inoculated plants at the end of the acquisition period (Day 11; Figure 3), nor on the epicuticle and internal bodies of *M. pygmaeus* and *N. tenuis* individuals that fed on the mock-inoculated plants (Day 14; Figure 4).

### 3.2. Beneficial Mirids Transmit Endophytic Bacteria by Feeding on Tomato Plants

*Macrolophus pygmaeus* and *N. tenuis* transmitted PsJN and 32A between tomato plants, and the bacterial strains were re-isolated from tomato shoots after mirid-mediated transmission (Day 14; Figure 3). In particular, 91.7% and 45.5% of tomato shoots showed a PsJN and 32A colonisation intensity of 1.16 × 10^5^ and 1.02 × 10^5^ CFU g^−1^ at the end of the *M. pygmaeus*-mediated transmission, respectively (Figure 3A). Likewise, 62.5% and 22.7% of the tomato roots showed a PsJN and 32A colonisation intensity of 2.40 × 10^2^ and 5.30 × 10^2^ CFU root^−1^ at the end of the *M. pygmaeus*-mediated transmission, respectively (Figure 3A). Moreover, the PsJN and 32A colonisation intensity was 7.72 × 10^4^ and 2.49 × 10^5^ CFU g^−1^ in 82.4% and 100% of tomato shoots or 5.09 × 10^3^ and 1.84 × 10^3^ CFU root^−1^ in 17.6% and 55.0% of tomato roots at the end of the *N. tenuis*-mediated transmission, respectively (Figure 3B). Moreover, PsJN and 32A were not detected in surface-disinfected mock-inoculated shoots and roots at the end of the mirid-mediated transmission (Figure 3A,B). Likewise, no colonies were detected in surface-disinfected mock-inoculated plants at the end of the acquisition period (sterility control; data not shown).

**Figure 5:**
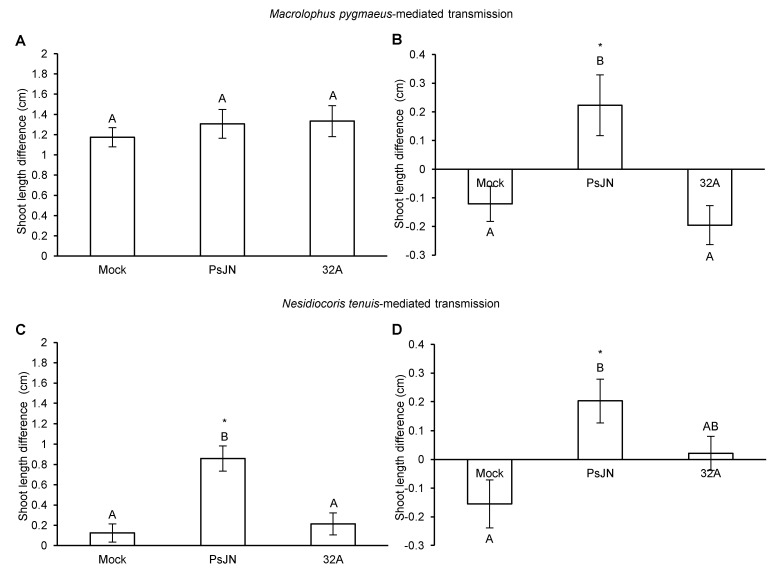
Effects of mirid feeding on tomato shoot length. Changes in shoot length caused by *Macrolophus pygmaeus* (**A**,**B**) or *Nesidiocoris tenuis* (**C**,**D**) feeding were assessed on mock-inoculated plants (Mock) and plants inoculated with *Paraburkholderia phytofirmans* PsJN (PsJN) or *Enterobacter* sp. 32A (32A) and calculated as the difference between the shoot length measured before (Day 7) and after (Day 11) the acquisition period (**A**,**C**), or before (Day 11) and after (Day 14) the mirid-mediated transmission (**B**,**D**). The two-way analysis of variance showed no significant differences between the two experimental repetitions (*p* > 0.05) and data from the two experiments were pooled. Mean and standard error values for positive samples and at least nine replicates (plants) are presented for each treatment. Different letters indicate significant differences among treatments according to Tukey’s test (*p* ≤ 0.05). Asterisks indicate significant differences in the pairwise comparisons between the mock-inoculated and bacterium-inoculated plants, according to Student’s *t*-test (*p* ≤ 0.05).

The difference in tomato shoot length measured at the beginning (Day 7) and at the end (Day 11) of *M. pygmaeus* feeding was comparable for the PsJN-, 32A- and mock-inoculated plants (Figure 5A), while that measured before (Day 11) and after (Day 14) *M. pygmaeus*-mediated transmission was greater in the PsJN-inoculated plants compared to mock- and 32A-inoculated plants (Figure 5B). The difference between tomato shoot length measured at the beginning and at the end of *N. tenuis* feeding (Figure 5C) and measured before and after *N. tenuis*-mediated transmission (Figure 5D) was greater in PsJN-inoculated plants compared to mock- and 32A-inoculated plants.

## 4. Discussion

Insects belonging to the suborder Heteroptera (e.g., mirids) are poorly studied as potential vectors compared to those belonging to the suborder Homoptera (e.g., leafhoppers, psyllids, aphids and whiteflies) [24]. Likewise, the significance of insects as vectors of plant pathogens is well characterised [54], while their role in the transmission of beneficial bacteria is less studied and in the case of mirids is unknown. This study assessed whether beneficial mirids, commonly used for biocontrol programs, could acquire and transmit beneficial microorganisms. Our results demonstrated that both mirids tested (*M. pygmaeus* and *N. tenuis*) are able to acquire the two beneficial bacterial strains (PsJN and 32A), both as bacteria adhering to the mirid epicuticle and in the mirid internal body through tissue contact and feeding on seed-inoculated tomato plants. Bacterial strains were visualised on the mirid abdomen, thorax and legs, but not in the mirid internal body, due to technical limitations related to the small size of mirids (3 mm). However, bacterial presence in the mirid internal body was confirmed by bacterial re-isolation from surface-disinfected mirids and 16S rRNA gene sequencing, supporting the mirid-mediated bacterial transmission from inoculated plants. This result indicates that tomato tissues are suitable for the acquisition of endophytic bacteria, likely because the PsJN and 32A strains are able to colonise the plant xylem [39,55]. For insect bacterial symbionts, such as *Cardinium* spp. [55] and *Wolbachia* spp. [56], transmission among plants has been demonstrated for homopteran insects (e.g., leafhoppers and whiteflies), but experimental evidence is limited for heteropteran insects [57]. In particular, bacterial isolates belonging to *Burkholderia* spp. were previously found in the midgut of bean bug (*Riptortus clavatus*, Hemiptera) and they were possibly acquired from the soil at the nymphal stage of bean bug [57], indicating the potential complexity of insect–microbe and microbe–plant mutualistic associations. Considering the mobile nature and piercing–sucking mouthparts of beneficial mirids, they could facilitate the introduction and spread of beneficial endophytes among crops [24,55], although further investigations under greenhouse or field conditions are required to validate the ecological reliability and applicative relevance of mirid-mediated transmission. It has been demonstrated that the two mirids have diverse feeding habits: *M. pygmaeus* feeds mainly on the mesophyll of leaves, stems and fruits, while *N. tenuis* feeds mainly within the vascular semi-ring on tomato plants [19,20], suggesting that the latter could be a more effective vector of microorganisms. In particular, 32A was transmitted by *N. tenuis* (100%) with higher efficacy compared to *M. pygmaeus* (45.5%), suggesting that mirids could selectively transmit plant endophytes, as previously described for *S. titanus* on grapevine [31]. Thus, further functional studies should be carried out for each combination of mirid species and bacterial strain, in order to better understand the possible crosstalk and species-specific interactions between mirids and beneficial bacteria that are responsible for selective transmission.

Successful plant colonisation is a crucial step for PGPB to promote growth [58]. In our study, beneficial endophytic bacteria were re-isolated from surface-disinfected tomato shoots and roots at the end of the mirid-mediated transmission at levels similar to seed inoculation, indicating their potential to establish stable colonies after mirid-mediated transmission. Plant colonisation is a complex mechanism and endophytes can establish colonies inside the plant root and then migrate to the stem [58]. Likewise, some endophytes can penetrate from the leaves either through natural openings or by vector transmission [59]. In our experiments, tomato roots were kept isolated from mirids by a paraffin layer, but they were colonised by PsJN and 32A, indicating that both strains systemically spread inside the plant after mirid-mediated transmission. Migration from stems to roots has already been demonstrated for 32A in grapevine [31]. In the case of increased predator density [5], mirid feeding on plants could lead to slight yield losses [16,17]. Mirids were provided with either mock-inoculated or bacterium-inoculated tomato plants as only food source and differences in tomato shoot length after *M. pygmaeus*-mediated transmission were greater in PsJN-inoculated plants compared to mock- and 32A-inoculated plants. Likewise, differences in tomato shoot length after *N. tenuis*-mediated transmission were greater in PsJN-inoculated plants compared to mock- and 32A-inoculated plants, indicating that PsJN could mitigate mirid feeding damage. These results may indicate that PGPB either reduce plant sensitivity to mirid feeding or influence plant development (e.g., nutrient provision and phytohormone production) to compensate for the mirid feeding damage, but the underlying mechanisms should be further elucidated. For example, some PGPB strains (e.g., *Pseudomonas putida* and *Rothia* spp.) can mitigate tobacco cutworm (*Spodoptera litura*) infestation in tomato [60], suggesting the potential use of PGPB to control arthropods and to stimulate plant growth [61].

In conclusion, our findings provide evidence of mirid-mediated transmission of beneficial bacterial strains and assessed the transmission efficacy, quantification and distribution pattern in tomato plant tissues (shoots and roots) and in mirid tissues (mirid epicuticle and internal body). In particular, PsJN and 32A can endophytically colonise tomato plants and move from shoots to roots after mirid-mediated transmission. In order to implement plant inoculation strategies with mirids, future steps must focus on the interactions between *M. pygmaeus* or *N. tenuis* and the PGPB strains in order to evaluate the persistence of beneficial bacteria on mirids. Possible unknown impacts of PGPB on mirid performance and efficacy as predators should be further investigated in order to verify PsJN and 32A transmission through feeding, physical contact, excretion and/or their combination. Further studies are also required to better characterise the molecular and functional interactions among mirids, bacteria and plants and to understand the mechanisms responsible for tissue colonisation and mirid-mediated transmission.

## Data Availability

All data obtained in this study can be found in the manuscript or in the supplementary materials.

## Supplementary Materials

The following are available online at https://www.mdpi.com/article/10.3390/microorganisms9061294/s1, Figure S1: Negative controls of double labelling of oligonucleotide probes for fluorescence in situ hybridisation.

## References

[1] Pertot I., Caffi T., Rossi V., Mugnai L., Hoffmann C., Grando M., Gary C., Lafond D., Duso C., Thiery D. (2017). A critical review of plant protection tools for reducing pesticide use on grapevine and new perspectives for the implementation of IPM in viticulture. Crop Prot..

[2] Redhead J.W., Powney G.D., Woodcock B.A., Pywell R.F. (2020). Effects of future agricultural change scenarios on beneficial insects. J. Environ. Manag..

[3] Calvo J., Bolckmans K., Stansly P.A., Urbaneja A. (2009). Predation by *Nesidiocoris tenuis* on *Bemisia tabaci* and injury to tomato. BioControl.

[4] Urbaneja A., Montón H., Mollá O. (2009). Suitability of the tomato borer *Tuta absoluta* as prey for *Macrolophus pygmaeus* and *Nesidiocoris tenuis*. J. Appl. Èntomol..

[5] Castañé C., Arnó J., Gabarra R., Alomar O. (2011). Plant damage to vegetable crops by zoophytophagous mirid predators. Biol. Control..

[6] Calvo F.J., Lorente M.J., Stansly P.A., Belda J.E. (2012). Preplant release of *Nesidiocoris tenuis* and supplementary tactics for control of *Tuta absoluta* and *Bemisa tabaci* in greenhouse tomato. Èntomol. Exp. Appl..

[7] Wakil W., Brust G.E., Perring T. (2018). Sustainable Management of Arthropod Pests of Tomato.

[8] Hobson G., Grierson D. (1993). Tomato.

[9] Gabarra R., Besri M. (1999). Tomatoes. Advances in Rice Blast Research.

[10] Gabarra R., AlOmar Ò., Castañé C., Goula M., Albajes R. (2004). Movement of greenhouse whitefly and its predators between in- and outside of Mediterranean greenhouses. Agric. Ecosyst. Environ..

[11] Put K., Bollens T., Wäckers F.L., Pekas A. (2012). Type and spatial distribution of food supplements impact population de-velopment and dispersal of the omnivore predator *Macrolophus pygmaeus* (Rambur) (Hemiptera: Miridae). Biol. Control.

[12] Moerkens R., Berckmoes E., Van Damme V., Wittemans L., Tirry L., Casteels H., De Vis R. (2017). Inoculative release strate-gies of *Macrolophus pygmaeus* Rambur (Hemiptera: Miridae) in tomato crops: Population dynamics and dispersal. J. Plant Dis. Prot..

[13] Schaefer C.W., Panizzi A.R. (2000). Heteroptera of Economic Importance.

[14] Perdikis D., Lykouressis D. (2000). Effects of Various Items, Host Plants, and Temperatures on the Development and Survival of *Macrolophus pygmaeus* Rambur (Hemiptera: Miridae). Biol. Control..

[15] Lykouressis D., Perdikis D., Michalaki M. (2001). Nymphal Development and Survival of *Macrolophus pygmaeus* Rambur (Hemiptera: Miridae) on Two Eggplant Varieties as Affected by Temperature and Presence/Absence of Prey. Biol. Control..

[16] Sánchez J.A., Lacasa A. (2008). Impact of the Zoophytophagous Plant Bug *Nesidiocoris tenuis* (Heteroptera: Miridae) on Tomato Yield. J. Econ. Èntomol..

[17] Sanchez J.A., López-Gallego E., Pérez-Marcos M., Perera-Fernández L.G., Ramírez-Soria M.J. (2018). How Safe Is It to Rely on *Macrolophus pygmaeus* (Hemiptera: Miridae) as a Biocontrol Agent in Tomato Crops?. Front. Ecol. Evol..

[18] Moerkens R., Berckmoes E., Van Damme V., Ortega-Parra N., Hanssen I.M., Wuytack M., Wittemans L., Casteels H., Tirry L., De Clercq P. (2015). High population densities of *Macrolophus pygmaeus* on tomato plants can cause economic fruit damage: Interaction with Pepino mosaic virus?. Pest Manag. Sci..

[19] Wheeler A.G. (2002). Biology of the Plant Bugs (Hemiptera: Miridae): Pests, Predators, Opportunists.

[20] Chinchilla-Ramírez M., Garzo E., Fereres A., Gavara-Vidal J., Broeke C.J.T., van Loon J.J., Urbaneja A., Pérez-Hedo M. (2021). Plant feeding by Nesidiocoris tenuis: Quantifying its behavioral and mechanical components. Biol. Control.

[21] Arnó J., Castañé C., Riudavets J., Roig J., Gabarra R. (2006). Characterization of damage to tomato plants produced by the zo-ophytophagous predator *Nesidiocoris tenuis*. IOBC/WPRS Bull..

[22] Moerkens R., Pekas A., Bellinkx S., Hanssen I., Huysmans M., Bosmans L., Wäckers F. (2020). *Nesidiocoris tenuis* as a pest in Northwest Europe: Intervention threshold and influence of Pepino mosaic virus. J. Appl. Èntomol..

[23] Perilla-Henao L.M., Casteel C.L. (2016). Vector-Borne Bacterial Plant Pathogens: Interactions with Hemipteran Insects and Plants. Front. Plant Sci..

[24] Gibb K.S., Randles J.W. (1991). Transmission of Velvet Tobacco Mottle Virus and Related Viruses by the Mirid Cyrtopeltis nicotianae. Advances in Disease Vector Research.

[25] Stahl F.J., Leupschen N.S. (1977). Transmission of *Erwinia amylovora* to pear fruti by *Lygus* spp. Plant Dis. Manag. Resport..

[26] Myhre E.A. (1988). Effects of *Lygus* spp. (Hemiptera: Miridae) on Mediterranean White Lupin (*Lupinus albus*). Ph.D. Thesis.

[27] Cooper W.R., Nicholson S.J., Puterka G.J. (2014). Potential transmission of *Pantoea* spp. and *Serratia marcescens* (Enterobacteriales: Enterobacteriaceae) to plants by *Lygus hesperus* (Hemiptera: Miridae). J. Econ. Èntomol..

[28] Gibb K.S., Randles J.W. (1988). Studies on the transmission of velvet tobacco mottle virus by the mirid, *Cyrtopeltis nicotianae*. Ann. Appl. Biol..

[29] Aramburu J., Galipienso L., Aparicio F., Soler S., López C. (2010). Mode of transmission of Parietaria mottle virus. J. Plant Pathol..

[30] Gibb K.S., Randles J.W. (1990). Distribution of velvet tobacco mottle virus in its mirid vector and its relationship to transmissibility. Ann. Appl. Biol..

[31] Lòpez-Fernàndez S., Mazzoni V., Pedrazzoli F., Pertot I., Campisano A. (2017). A Phloem-Feeding Insect Transfers Bacterial Endophytic Communities between Grapevine Plants. Front. Microbiol..

[32] Davison J. (1988). Plant Beneficial Bacteria. Nat. Biotechnol..

[33] Ferreira C.M., Soares H., Soares E.V. (2019). Promising bacterial genera for agricultural practices: An insight on plant growth-promoting properties and microbial safety aspects. Sci. Total Environ..

[34] Gaiero J.R., McCall C.A., Thompson K.A., Day N.J., Best A.S., Dunfield K.E. (2013). Inside the root microbiome: Bacterial root endophytes and plant growth promotion. Am. J. Bot..

[35] Sessitsch A., Coenye T., Sturz A.V., Vandamme P., Barka E.A., Salles J.F., Van Elsas J.D., Faure D., Reiter B., Glick B.R. (2005). *Burkholderia phytofirmans* sp. nov., a novel plant-associated bacterium with plant-beneficial properties. Int. J. Syst. Evol. Microbiol..

[36] Sawana A., Eadeolu M., Gupta R.S. (2014). Molecular signatures and phylogenomic analysis of the genus *Burkholderia*: Proposal for division of this genus into the emended genus *Burkholderia* containing pathogenic organisms and a new genus *Paraburkholderia gen.* nov. harboring environmental species. Front. Genet..

[37] Pillay V.K., Nowak J. (1997). Inoculum density, temperature, and genotype effects on in vitro growth promotion and epiphytic and endophytic colonization of tomato (*Lycopersicon esculentum* L.) seedlings inoculated with a pseudomonad bacterium. Can. J. Microbiol..

[38] Sharma V.K., Nowak J. (1998). Enhancement of verticillium wilt resistance in tomato transplants by in vitro co-culture of seedlings with a plant growth promoting rhizobacterium (*Pseudomonas* sp. strain PsJN). Can. J. Microbiol..

[39] Galambos N., Compant S., Moretto M., Sicher C., Puopolo G., Wäckers F., Sessitsch A., Pertot I., Perazzolli M. (2020). Humic Acid Enhances the Growth of Tomato Promoted by Endophytic Bacterial Strains Through the Activation of Hormone-, Growth-, and Transcription-Related Processes. Front. Plant Sci..

[40] Campisano A., Pancher M., Puopolo G., Puddu A., López-Fernández S., Biagini B., Yousaf S., Pertot I. (2014). Diversity in Endophyte Populations Reveals Functional and Taxonomic Diversity between Wild and Domesticated Grapevines. Am. J. Enol. Vitic..

[41] Lòpez-Fernàndez S., Compant S., Vrhovsek U., Bianchedi P.L., Sessitsch A., Pertot I., Campisano A. (2015). Grapevine colonization by endophytic bacteria shifts secondary metabolism and suggests activation of defense pathways. Plant Soil.

[42] Sanchez J.A., Lacasa A., Arno J., Castañé C., AlOmar O. (2009). Life history parameters for *Nesidiocoris tenuis* (Reuter) (Het., Miridae) under different temperature regimes. J. Appl. Èntomol..

[43] Schneider C.A., Rasband W.S., Eliceiri K.W. (2012). NIH Image to ImageJ: 25 years of image analysis. Nat. Methods.

[44] Boucher M., Collins R., Harling K., Brind’Amour G., Hesler S., Wentworth K., Cox K., Loeb G. (2021). Field Evaluation of Interactions between Insects and *Erwinia amylovora* in a New York Apple Orchard. PhytoFrontiers™.

[45] Zhu Y., Kawai H., Hashiba S., Amarasiri M., Kitajima M., Okabe S., Sano D. (2020). The Effect of GD1a Ganglioside-Expressing Bacterial Strains on Murine Norovirus Infectivity. Molecules.

[46] Kumar S., Stecher G., Li M., Knyaz C., Tamura K. (2018). MEGA X: Molecular evolutionary genetics analysis across computing platforms. Mol. Biol. Evol..

[47] Compant S., Mitter B., Colli-Mull J.G., Gangl H., Sessitsch A. (2011). Endophytes of Grapevine Flowers, Berries, and Seeds: Identification of Cultivable Bacteria, Comparison with Other Plant Parts, and Visualization of Niches of Colonization. Microb. Ecol..

[48] Amann R., Binder B.J., Olson R.J., Chisholm S.W., Devereux R., Stahl D. (1990). Combination of 16S rRNA-targeted oligonucleotide probes with flow cytometry for analyzing mixed microbial populations. Appl. Environ. Microbiol..

[49] Daims H., Brühl A., Amann R., Schleifer K.-H., Wagner M. (1999). The Domain-specific Probe EUB338 is Insufficient for the Detection of all Bacteria: Development and Evaluation of a more Comprehensive Probe Set. Syst. Appl. Microbiol..

[50] Mitter B., Pfaffenbichler N., Flavell R., Compant S., Antonielli L., Petric A., Berninger T., Naveed M., Sheibani-Tezerji R., Von Maltzahn G. (2017). A New Approach to Modify Plant Microbiomes and Traits by Introducing Beneficial Bacteria at Flowering into Progeny Seeds. Front. Microbiol..

[51] Manz W., Amann R., Ludwig W., Wagner M., Schleifer K.-H. (1992). Phylogenetic Oligodeoxynucleotide Probes for the Major Subclasses of Proteobacteria: Problems and Solutions. Syst. Appl. Microbiol..

[52] Wallner G., Amann R., Beisker W. (1993). Optimizing fluorescent in situ hybridization with rRNA-targeted oligonucleotide probes for flow cytometric identification of microorganisms. Cytometry.

[53] Hammer Ø., Harper D.A.T., Ryan P.D. (2001). PAST: Paleontological statistics software package for education and data analysis. Palaeontol. Electron..

[54] Heck M. (2018). Insect Transmission of Plant Pathogens: A Systems Biology Perspective. mSystems.

[55] Gonella E., Pajoro M., Marzorati M., Crotti E., Mandrioli M., Pontini M., Bulgari D., Negri I., Sacchi L., Chouaia B. (2015). Plant-mediated interspecific horizontal transmission of an intracellular symbiont in insects. Sci. Rep..

[56] Li S.-J., Ahmed M.Z., Lv N., Shi P.-Q., Wang X.-M., Huang J.-L., Qiu B.-L. (2017). Plantmediated horizontal transmission of Wolbachia between whiteflies. ISME J..

[57] Kikuchi Y., Hosokawa T., Fukatsu T. (2007). Insect-Microbe Mutualism without Vertical Transmission: A Stinkbug Acquires a Beneficial Gut Symbiont from the Environment Every Generation. Appl. Environ. Microbiol..

[58] Compant S., Clément C., Sessitsch A. (2010). Plant growth-promoting bacteria in the rhizo- and endosphere of plants: Their role, colonization, mechanisms involved and prospects for utilization. Soil Biol. Biochem..

[59] Frank A.C., Guzmán J.P.S., Shay J.E. (2017). Transmission of Bacterial Endophytes. Microorganisms.

[60] Bano A., Muqarab R. (2017). Plant defence induced by PGPR againstSpodoptera liturain tomato (Solanum lycopersicumL.). Plant Biol..

[61] Ruiu L. (2020). Plant-Growth-Promoting Bacteria (PGPB) against Insects and Other Agricultural Pests. Agronomy.

