# Supplementary material for: Beneficial Insects Deliver Plant Growth-Promoting Bacterial Endophytes between Tomato Plants
Source: Microorganisms. 2021 Jun 14;9(6):1294. doi: 10.3390/microorganisms9061294 (PMC8231829; doi:10.3390/microorganisms9061294)
Supplement: Supplementary file 1 [file microorganisms-09-01294-s001.zip › microorganisms-1248722-supplementary.pdf]

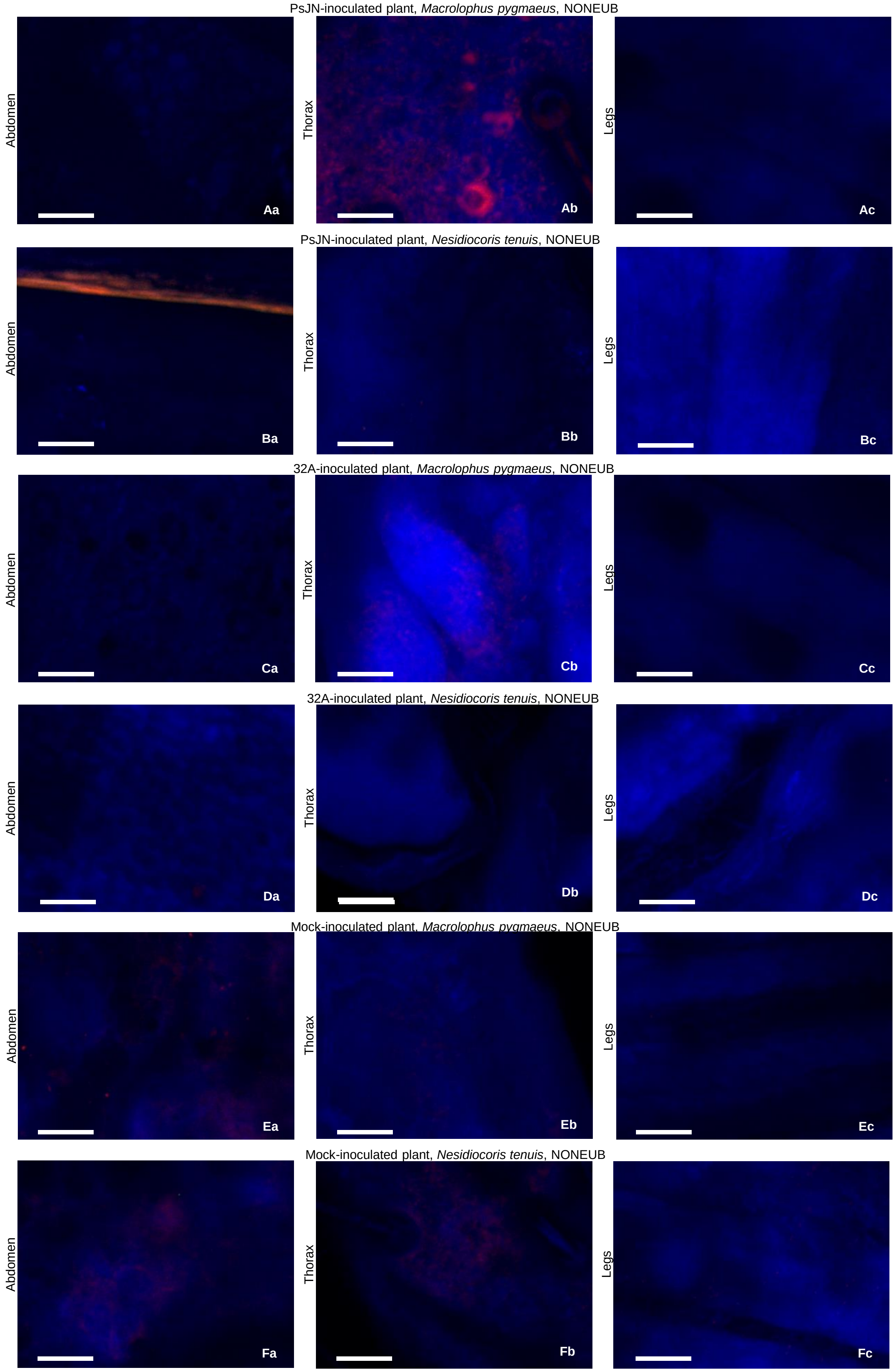

Figure S1. Negative controls of double labelling of oligonucleotide probes for fluorescence *in situ* hybridisation. *Macrolophus pygmaeus* (A, C and E) and *Nesidiocoris tenuis* (B, D and F) abdomen (a), thorax (b) and legs (c) samples were collected four days after feeding (acquisition period) on plants inoculated with *Paraburkholderia phytofirmans* PsJN (PsJN) (A, B) or *Enterobacter* sp. 32A (32A) (C, D) or mock-inoculated plants (E, F) and hybridised with the NONEUB probe as negative probe not targeting bacterial sequences. Five replicates (plants) were analysed for each treatment and representative pictures were selected. Bars correspond to 10 µm.
